# Psychometric properties of the Trauma Checklist 2.0 and its predictive utility of felony re-offending among high-risk juvenile offenders

**DOI:** 10.1186/s13034-023-00656-1

**Published:** 2023-09-21

**Authors:** Jenna N. Shold, J. Michael Maurer, Brooke L. Reynolds, Aparna R. Gullapalli, Corey H. Allen, Bethany G. Edwards, Nathaniel E. Anderson, Carla L. Harenski, Craig S. Neumann, Kent A. Kiehl

**Affiliations:** 1 The Mind Research Network, 1101 Yale Boulevard NE, Albuquerque, NM 87106-4188 USA; 2 School of Graduate Psychology, Pacific University, 705 SE Baseline Street, Hillsboro, OR 97123 USA; 3 Department of Psychology, University of New Mexico, 1 University of New Mexico, Albuquerque, NM 87131-0001 USA; 4 Department of Psychology, University of North Texas, 1155 Union Circle 311280, Denton, TX 76203-5017 USA; 5 Departments of Psychology, Neuroscience, and Law, University of New Mexico, 1 University of New Mexico, Albuquerque, NM 87131-0001 USA

**Keywords:** Childhood trauma, Assessment development, Psychometrics, Re-offense prediction

## Abstract

**Background:**

Incarcerated youth are characterized by particularly high rates of childhood trauma, a significant risk factor for outcomes including risky behaviors and recidivism. Trauma-based interventions can ameliorate the negative effects of childhood trauma; however, a critical part of success is careful trauma screening. Due to the limitations associated with commonly used self-report trauma assessments, our team developed the Trauma Checklist (TCL), a trained-rater assessment of childhood trauma specifically created for use with forensic populations. The TCL is designed to provide a more comprehensive assessment of trauma, incorporating categories that are of specific relevance for incarcerated individuals (e.g., traumatic loss). Here, we discuss the continued development made to our original trauma assessment and explore the psychometric properties of this expanded assessment (herein termed the TCL 2.0).

**Method:**

We examined relationships between TCL 2.0 scores, measures of psychopathology, and psychopathic traits in a sample of incarcerated male juvenile offenders (*n* = 237). In addition, we examined whether TCL 2.0 scores were associated with time to felony re-offense via Cox proportional-hazard regression analyses.

**Results:**

We examined dimensionality of the TCL 2.0 using a principal component analysis (PCA), the results of which were confirmed via exploratory structural equation modeling; the PCA yielded a two-component solution (i.e., PC1 and PC2). We observed that PC1 (Experienced Trauma) scores were positively correlated with mood disorder diagnoses. TCL 2.0 total scores were positively correlated with post-traumatic stress disorder symptomatology and psychopathic traits. Finally, higher PC2 (Community Trauma) scores were associated with faster time to felony re-offending.

**Conclusions:**

These results suggest that the TCL 2.0 may be a beneficial screening tool to provide high-risk youth with appropriate trauma-informed treatment.

## Background

Childhood trauma typically involves experiencing or witnessing an event that causes lasting psychological or physical harm to the individual [1]. Because these events are usually unexpected, an individual may experience lingering feelings of vulnerability and helplessness as a result [2]. One method for studying childhood trauma is to examine the rates of adverse childhood experiences (ACEs), traumatic events that can happen to children [3]. While ACEs do not necessarily capture all aspects related to an individual’s childhood trauma, commonly studied ACEs include psychological abuse, physical abuse, sexual abuse, living with a caretaker with mental health or substance use problems, witnessing violence towards one’s mother, and living with someone who went to prison [4]. Approximately 50% of children in the United States (US) report at least one ACE and over 20% report two or more such experiences [5]. Among incarcerated youth, the rates of ACEs are considerably higher. For example, one study examining ACEs in a large sample of juvenile offenders in the US found that over 95% of the sample reported at least one ACE, and over 50% reported four or more ACEs [6]. Recent studies have reported similar childhood trauma prevalence rates among juvenile offenders worldwide [7–10]. Due to the exceptionally high rates of childhood trauma experienced by juveniles involved in the criminal justice system, it is imperative to assess childhood trauma thoroughly and accurately to provide these youth with the appropriate trauma-based interventions.

Childhood trauma is associated with significant negative health outcomes, including ischemic heart disease, cancer, chronic lung disease, skeletal fractures, and liver disease [4]. In addition, childhood trauma has been linked to increased incidence of alcohol and substance use, eating disorders, suicide ideations and attempts, high-risk sexual behavior, sleep disturbances, depression, and post-traumatic stress disorder (PTSD) [4, 11–16]. Exposure to childhood trauma has also been associated with self-destructive and risky behavior (sometimes referred to as post-traumatic risk-seeking), as well as involvement with the criminal justice system [17, 18]. When comparing individuals who were exposed to childhood trauma to non-trauma exposed control subjects, those with childhood trauma exposure were over four times more likely to be arrested during adolescence and twice as likely to be arrested during adulthood [19]. Childhood maltreatment has emerged as a strong predictor of recidivism [20, 21], and the risk of becoming a serious, violent, and chronic offender increases as the number of ACEs increases [22]. However, the effects of at least some of these negative outcomes may be ameliorated with proper trauma-based intervention. For example, such interventions have been shown to reduce rates of PTSD and substance use disorders, and are associated with improved coping skills [23, 24]. In addition, trauma-informed care for youth involved in the criminal justice system reduces problem behaviors as well as delinquency [25]. A core aspect of successful trauma-based interventions is the trauma screening component, which allows treatment staff to target appropriate intervention strategies [26]. If treatment providers do not have a full understanding of the types of trauma and experiences of their patients, there could be a tendency to downplay highly significant traumatic events [27]. Because childhood trauma is linked with many negative health and behavioral outcomes, implementation of trauma-informed interventions may help to ameliorate outcomes in high-risk youth; however, successful implementation of such interventions requires a reliable and valid tool with which to assess exposure to childhood trauma.

Assessments have been developed to measure childhood trauma, including the commonly used Childhood Trauma Questionnaire (CTQ) and the Juvenile Victimization Questionnaire (JVQ) [28, 29]. The CTQ is a 28-item self-report measure assessing severity of emotional abuse and neglect, physical abuse and neglect, and sexual abuse [28]. The JVQ assesses 34 offenses against youth covering five general areas, including conventional crime, childhood maltreatment, peer and sibling victimization, sexual victimization, and witnessing/indirect victimization. The JVQ can be administered by interviewing the youth or their caretaker, and is also available in a self-administered format [29]. However, these existing trauma assessments have some limitations, especially when assessing childhood trauma among incarcerated offenders. Specifically, the CTQ and JVQ were not designed for use in forensic populations [28, 30], and therefore do not assess all potentially relevant trauma-related experiences. For example, the CTQ and JVQ do not assess loss of a parent or caretaker during childhood, even though this variable has been associated with increased criminal behavior during adulthood [31, 32]. Importantly, as these existing trauma measures were developed in non-incarcerated individuals, they are not designed to account for the substantially high rates of childhood trauma that characterize incarcerated individuals. Relatedly, existing trauma assessments often assess childhood trauma exposure more broadly, and in doing so, they may not capture full information about an incarcerated individual’s childhood trauma history. For these reasons, it can be difficult to fully examine the impact of childhood trauma in forensic populations using such existing childhood trauma assessments. In addition, accurate completion of self-report assessments or interviews relies entirely on the cooperation of the individual, which could limit the information gathered in the case of a reluctant participant. Individuals in correctional institutions are often characterized by reading comprehension difficulties [33]; as such, self-report measures of trauma may be difficult for incarcerated individuals to understand and complete correctly and accurately. Furthermore, incarcerated individuals may underreport trauma experiences for a variety of reasons, including mistrust of providers or researchers, feelings of guilt or self-blame, feeling the need to maintain privacy in order to limit vulnerability within correctional settings, and fear of negative consequences [34, 35]. Incarcerated participants may also lack proper insight and judgement needed to complete trauma-related self-report assessments or interviews, due to the normalization of their childhood experiences [36]. On the other hand, trauma could also be over-reported via self-report measures, in order to gain sympathy, to feel more important or powerful, and to attempt to gain a better outcome in their legal case [37, 38]. These issues emphasize the need for childhood trauma assessments to have additional, objective information with which to accurately assess trauma history and to better inform trauma-based treatment.

To overcome these obstacles and to provide a thorough assessment of childhood trauma, our research group has developed a trained-rater assessment specifically for use in forensic settings [39]. The Trauma Checklist (TCL) relies on trained raters who score individual participants’ childhood trauma experiences across seven categories, including emotional abuse, physical abuse, sexual abuse, neglect and poverty, community violence, observed trauma, and traumatic loss. Because the TCL was designed for use in forensic populations, it includes additional trauma categories that are of particular relevance to incarcerated individuals who report higher levels of childhood trauma [6–10], including traumatic loss (i.e., death of a family member or close friend, willful abandonment, and/or separation from a parent or caregiver). To address issues surrounding cooperation, reading comprehension difficulties, clinical insight, and overreporting or underreporting trauma history, the TCL assessment was designed such that trained raters review and score trauma experiences using multiple sources, including clinical measures of personality and psychopathology, as well as objective information available in participants’ institutional files. Collecting trauma-related information from multiple sources, combined with trained raters scoring the TCL assessment, aids in providing the most accurate and comprehensive scoring of trauma-related items for each individual assessed. In our initial development of the TCL assessment [39], TCL total scores were positively correlated with PTSD symptomatology and CTQ total scores. Overall, these findings suggest that the TCL assessment has a high degree of utility in assessing childhood trauma, while also incorporating additional trauma categories that are specifically relevant in high-risk forensic populations (i.e., traumatic loss).

Here we developed a modified and expanded version of our original trauma assessment, hereby referred to as the TCL 2.0. Alterations were made to our original TCL assessment to assess additional information relating to an individual’s childhood trauma experiences. For example, with the TCL 2.0, additional resources were used to assess childhood trauma. In our original TCL assessment, resources including the participant’s institutional file and demographic information were used for scoring, as well as additional instruments, including the Psychopathy Checklist: Youth Version (PCL:YV) [40], Post-Head Injury Symptoms Questionnaire [41], and the Massachusetts Youth Screening Instrument-Version 2 (MAYSI-2; [42]). Similar assessments were used to score the TCL 2.0, and some additional instruments were used to gather more comprehensive trauma information for scoring purposes, including the Upsetting Events Survey (UES; [43]), My Worst Experience Scale [44], Childhood PTSD Symptoms Scale (CPSS; [45]), Kiddie-Schedule for Affective Disorders and Schizophrenia (K-SADS; [46]), CTQ [47], and Socioeconomic Status Questionnaire (SES Questionnaire; [48]). Additionally, to assist raters with scoring criteria, our modified TCL 2.0 assessment has included more detailed instructions and examples for how to score childhood trauma (i.e., general guidance regarding how to score as well as common types of experiences that should be considered in scoring). Furthermore, rather than having an overall trauma score for each trauma category, in the TCL 2.0 we have added age bins to assess trauma occurring from ages 0–6, 7–12, and 13–18 for each of the seven trauma categories. With the addition of the age bin scoring within each trauma category, the TCL 2.0 allows for the calculation of *chronicity* scores in addition to TCL 2.0 total scores that were included in our original trauma assessment. The chronicity score calculation provides a more comprehensive measure of trauma severity. This allows us to distinguish between individuals who experienced singular traumatic events during their childhood versus those who experienced chronic, long-term traumatic events.

Due to the modifications to our original TCL instrument, the aims of the current study were to provide details on the expansions included within the TCL 2.0, to examine correlations between the TCL 2.0 and variables related to childhood trauma, and to examine the predictive utility of the TCL 2.0 for high-risk antisocial outcomes. First, we describe in extended detail the construction and development of the TCL 2.0. Furthermore, we report correlations between the TCL 2.0 and measures of depression, anxiety, PTSD, and psychopathic traits, consistent with our previously published report [39]. Finally, expanding upon our original report, we investigate whether TCL 2.0 scores were significantly associated with re-offending outcomes, specifically, predicting time (in months) to felony re-offense via Cox proportional-hazard regression analyses. Previous studies suggest that childhood trauma is a significant predictor of recidivism [20, 21]. Here, we investigate whether the TCL 2.0 would aid in the prediction of future antisocial outcomes, even when controlling for other variables previously associated with re-offending, including intelligence quotient (IQ) scores and psychopathic traits [49, 50].

## Method

### Sample

The TCL 2.0 was archivally scored in 2020–2021 using data collected from juvenile offenders housed at a maximum-security juvenile correctional facility in New Mexico. These individuals had previously participated in a NIMH-funded research study (R01 MH071896); data collection for this study occurred from the years 2007 to 2011. The final sample (*n* = 237 males) consisted of participants with full data collection completed. Consistent with our prior report [39], we used only males in our current sample; however, this sample is different from our previous report (i.e., this report includes juveniles who were incarcerated at a maximum-security correctional facility in New Mexico, whereas our previous report included juveniles incarcerated at a treatment center in Wisconsin). A sample of incarcerated offenders was chosen for investigation, as incarcerated youth are characterized by considerably high rates of trauma. For example, one report found that 86% of adolescents in the New Mexico juvenile justice system experienced four or more ACEs [51], making this an ideal sample for archivally assessing trauma using the TCL 2.0. Participants included in the final sample ranged from 14.08 to 21.17 years of age (*M* = 17.79; *SD* = 1.15). Full-scale IQ was estimated from Vocabulary and Matrix Reasoning subtests using the Wechsler Adult Intelligence Scale—3rd Edition (WAIS-III; [52]) for participants sixteen years of age or older and from the Wechsler Intelligence Scale for Children—4th Edition (WISC-IV; [53]) for participants younger than sixteen years of age. IQ estimates for this sample ranged from 63 to 140 (*M* = 92.18; *SD* = 12.06). In our sample, 76.79% of participants reported to be Hispanic or Latino, 21.94% reported to be non-Hispanic or Latino, and 1.27% chose not to report their ethnicity. Racial breakdown of the sample was as follows: 11.39% American Indian/Alaskan Native, 3.38% Black or African American, 0.42% Native Hawaiian or other Pacific Islander, 60.76% White, 7.59% reported more than one race, and 16.46% chose not to report their race.

### Study Procedures & Ethics

Research staff recruited participants by making announcements and sharing information about the study throughout the juvenile correctional facility. Research staff obtained informed consent from all study participants over the age of 18 and informed assent, along with their parent or legal guardian’s informed consent, for participants under the age of 18. Participants were paid at an hourly rate that was comparable to the current institutional wage for general labor. Procedures for the study were approved by the University of New Mexico Human Research Review Committee, the Office for Human Research Protections (OHRP), and by the staff at the juvenile correctional facility where the study was conducted.

Private rooms at the facility were used to conduct interviews and administer assessments to study participants. Two interviews were completed (i.e., the PCL: YV and K-SADS), and participants agreed to have these interviews videotaped. Video recordings were performed so that research staff working on the overall study could review the recordings for the purposes of training and double-rating. These interviews, in addition to other study assessments and the participant’s criminal and institutional records, were used to archivally score each participant on the TCL 2.0.

### TCL 2.0

Trauma scoring was completed using the TCL 2.0, which contains several expansions compared to our original trauma scoring assessment (for more detailed information regarding the original trauma scoring completed by our research group, see [39]). A score of zero, one, or two was assigned to each trauma category (see “TCL 2.0 Scoring” section for more details). While both scoring methods used the same seven categories of trauma, expansions to the TCL 2.0 include more detailed definitions and examples under each category of abuse as well as raters using additional assessments to score the TCL 2.0. In addition, guidance was provided to raters on how to score commonly reported trauma information. Regarding use of institutional file information, our original TCL assessment relied heavily on reports available in the participant’s institutional file to confirm abuse history for scoring purposes (e.g., to receive a score of two, the institutional file had to confirm the participant’s account of the abuse). However, due to the varying level of detail regarding childhood trauma in each participant’s file and the fact that institutional records do not always include information about certain types of traumatic experiences, this criterion was removed from the TCL 2.0 (e.g., a participant could score a two even if reported abuse was not found in the participant’s institutional record). Finally, the original TCL scoring only assigned total trauma scores for each specific trauma category, whereas the TCL 2.0 includes scores for each age bin (i.e., from years 0–6, 7–12, and 13–18) under each trauma category in addition to the total scores for each trauma category. This allows raters to calculate the severity of trauma history (i.e., a chronicity score).

### Training of Raters

Research staff with at least a bachelor’s degree in psychology or a related field scored the TCL 2.0 assessment. A research staff member who was experienced in scoring the TCL 2.0 assessment trained the raters. Staff were given the instructions and scoring sheet for the TCL 2.0, and they independently rated an assigned study participant on the TCL 2.0 using the provided instructions. After all staff had independently rated the assigned participant, an experienced TCL 2.0 rater extensively reviewed the scoring with all staff for the assigned participant. All TCL 2.0 scoring questions were answered during this session, and the experienced research staff member ensured that all raters were knowledgeable in the scoring of the TCL 2.0 before staff began to score participants.

### Double Rating

Research staff were paired with another research staff member and given a list of study participants to score on the TCL 2.0. Each research staff member independently rated their list of participants and then met with their partner (i.e., another research staff member) to come to consensus on final scores for each study participant. For this sample, *n* = 233 study participants were independently scored by two raters and final TCL 2.0 scores were determined at a consensus meeting between the two raters assigned to that participant. Four remaining study participants were single-rated by an experienced staff member.

### Categories of Abuse

Trauma was rated by categorizing experiences (as recorded in interviews, assessments, or the institutional file) into one of seven types of abuse. The seven trauma categories of the TCL 2.0, in addition to their definitions, are as follows:

#### Emotional Abuse

Adults in the family or someone acting in a caregiver role behaving in a way that implies that they do not care about the child. Statements intended to make the child feel bad, embarrassed, or humiliated were included under this item, as were instances of exploitation by the parent/caregiver, instances of the participant being threatened with physical violence or forced to do something against their will, and emotional manipulation.

#### Physical Abuse

Adults in the family, someone acting in a caregiver role, or a figure of authority physically harming the child. Reports of the participant being hit, pushed, kicked, or involved in any other physical altercation which left bruises or marks were included.

#### Sexual Abuse

Anyone forcing the child to do something sexual against their will. Due to high frequency of participants reporting that they had consensual sexual relationships in their teens with someone over the age of 18, relationships reported as consensual were not counted in this item. However, if the participant had a consensual sexual relationship with someone who was four or more years older than them, raters did make note of this.

#### Neglect/Poverty

Inability or refusal of the child’s caregiver to provide safety and care. Also included in this item was the inability or refusal of the caretaker to provide support and love to the child (e.g., refusal to visit child during incarceration and/or treatment, or caregivers making and breaking promises to the child). Poverty was included only if it resulted in a neglectful environment.

#### Community Violence

Exposure to neighborhood factors that might cause physical or psychological harm. Included in this item were things such as exposure to violence via gang affiliations, being the victim of a violent crime, physical abuse by peers, and frequent exposure to general violence in the child’s neighborhood or community.

#### Observed Trauma

Witnessing acts of violence against another person, including domestic and community violence. This item also includes witnessing a negative consequence of a parent/caregiver using illicit drugs or alcohol (e.g., child witnessing a parent/caregiver having a seizure or overdosing on a substance).

#### Traumatic Loss

Experiencing the death of a family member or close friend, willful abandonment, and/or traumatic separation from a parent or caregiver for an extended period of time (e.g., police arrest of a parent at gunpoint in front of the child, parent imprisonment where the other parent could not adequately care for the child).

### TCL 2.0 Scoring

A score of zero, one, or two was used to rate experiences under each trauma category. Raters assigned a score of a zero when the participant denied having been abused and when no evidence of abuse was found during interviews and file review. A score of one was assigned when there was one or only a few minor instances of abuse detailed in the institutional file, assessments, or interviews. A score of two was assigned when either the institutional file, assessments, or interviews contained one serious instance of abuse or abuse that occurred over a long period of time.

Each trauma category was broken down into three age bins (0–6, 7–12, and 13–18 years old) as well as a total rating for each trauma category. A score of zero, one, or two was given for each age bin, as well as for each category’s total rating. If a score of zero was assigned to all three age bins for a particular category, a participant would receive a total score of zero for that specific trauma category. If a participant received a score of one in any age bin for a category, then they would receive a total score of one for that trauma category. If a participant received a score of two in any age bin for a category, then they would receive a total score of two for that trauma category, regardless of the presence of zeros or ones in other age bins. In instances where a participant received a one across all age bins for a category, raters gave a total trauma rating of a one or a two for that category, depending on abuse severity.

### Calculation of Scores

For each participant, a total score and a chronicity score was calculated for the TCL 2.0. The TCL 2.0 total score was calculated by adding the total scores for each of the seven trauma categories. Possible TCL 2.0 total scores range from 0 to 14. The total chronicity score was calculated by summing the values in all age bins for each of the seven categories, with total chronicity scores ranging from 0–42.

### Information Used for TCL 2.0 Scoring

The participant’s institutional file, as well as several other assessments, were used for scoring the TCL 2.0. Importantly, when assessments were used for TCL 2.0 scoring (e.g., PCL:YV and K-SADS), notes regarding traumatic experiences were used and this was separate from scoring of psychopathic traits and symptoms of psychopathology.

#### Institutional File

Criminal files were reviewed in detail by raters in order to have objective information with which to score the TCL 2.0. While information within each participant’s file varied, all criminal files included at least some of the following reports: psychiatric reports, risk assessments, competency assessments, social services reports, social histories completed by social workers, child protective services reports, education reports, and law enforcement incident reports.

In addition to reviewing the participant’s institutional file, research staff also asked a series of questions after the participant consented to being part of the study. Information regarding prior surgeries and metal screening, health and medical history, and incarceration history was gathered. Review of any prior surgery and metal screening questions (e.g., Have you ever been injured with a metallic object?) were particularly useful for scoring the TCL 2.0.

#### Psychopathy Checklist: Youth Version (PCL:YV)

The PCL:YV was used to assess psychopathic traits. The PCL:YV consists of 20 psychopathic traits (e.g., pathological lying, lack of remorse, criminal versatility) which the interviewer rates on a scale of 0–2, depending on the degree to which the item applies to the participant [40]. Ratings are based on information collected during a semi-structured interview as well as an extensive review of the participant’s criminal record. Psychopathic traits fall into four facets, which are nested under two factors [54]. Facet 1, which consists of interpersonal traits (conning and manipulative behavior and grandiosity), and Facet 2, which consists of affective traits (callousness and shallow affect), both fall under Factor 1. Facet 3, which consists of lifestyle traits (irresponsibility and impulsivity), and Facet 4, which consists of antisocial/developmental traits from childhood through adulthood (juvenile delinquency and criminal versatility), fall under Factor 2.

Previous studies have reported associations between childhood trauma and psychopathic traits in juvenile and adult offenders [55–57]. Because the PCL:YV interview provides extensive details regarding the participant’s life, this assessment was used for TCL 2.0 scoring. Raters specifically reviewed the sections pertaining to family life, peer/sexual relationships, school history and adjustment, and childhood/adolescent antisocial behaviors for information relating to traumatic life events. Assessment notes and videotaped PCL:YV interviews were reviewed for TCL 2.0 scoring purposes.

#### Kiddie-Schedule for Affective Disorders and Schizophrenia (K-SADS)

The K-SADS was used to assess psychopathological diagnoses among study participants. This semi-structured interview is commonly used to diagnose psychopathology in children aged 6–18, including depressive disorders, psychotic disorders, trauma-related disorders, conduct disorders, and substance-related disorders [46]. TCL 2.0 raters reviewed the K-SADS assessment for endorsement of traumatic events included in the PTSD checklist, as well as notes taken during the interview by the K-SADS rater (e.g., notations regarding family death, community violence, and home life were often included in the K-SADS rater notes, which were in turn used for TCL 2.0 scoring). In addition, raters listened to the videotaped K-SADS interviews to obtain more detailed information regarding the participant’s childhood trauma. While the entire K-SADS was used for scoring, information from the mood and trauma-related sections were particularly useful with regard to TCL 2.0 scoring.

#### Post-Head Injury Symptoms Questionnaire (PHQ)

The PHQ asks about head injuries that participants have incurred and the symptoms associated with each injury [41]. TCL 2.0 raters looked at the events surrounding the head injury to gather information on trauma (e.g., if the participant reported they were hit over the head by a parent or guardian, this would count as physical abuse on the TCL 2.0).

#### Upsetting Events Survey (UES)

This assessment asks 17 questions regarding upsetting events that sometimes happen to people [43]. Potential responses for each question include: “No”, “Yes”, “More than one time”, or “I don’t know.” For two questions, the participants were asked to write down the event they were thinking of when they answered the question. Raters reviewed each participant’s endorsement of traumatic experiences to score the TCL 2.0.

#### Childhood PTSD Symptoms Scale (CPSS)

The CPSS assesses PTSD diagnosis and severity of symptoms in individuals aged 8–18. It asks about all *DSM-IV* PTSD symptoms and their prevalence as well as any functional impairment that resulted from the upsetting event [45]. For this assessment, raters reviewed information to score the TCL 2.0.

#### Childhood Trauma Questionnaire (CTQ)

This self-report questionnaire asks participants to respond to questions about their experiences growing up as a child and teenager on a 5-point scale [47]. Staff rating the TCL 2.0 reviewed answers to questions regarding emotional, physical, sexual, and neglectful experiences that the participant may have endorsed. Because the CTQ does not gather thorough detail on childhood trauma, additional trauma information was gathered from more detailed sources for TCL 2.0 scoring purposes (e.g., K-SADS, PCL:YV, institutional files). In the current sample, the CTQ was not administered while participants were incarcerated as youth. Instead, for a subsample of participants (*n* = 49), the CTQ was collected as part of a large follow-up study (R01 HD092331) later as adults.

#### My Worst Experience Scale

This two part self-report questionnaire asks the participant about the worst experience they have ever had [44]. Part I asks the participant six questions to ascertain more details about the specific experience, and Part II asks participants 105 questions to describe what happened after their worst experience (responses on a six-point scale, with additional questions about frequency). Raters reviewed Part I of the assessment, which describes the event in question, for TCL 2.0 scoring.

#### Socioeconomic Status (SES) Questionnaire

This questionnaire asks the participant about marital status, education and work history, living arrangements, and parental education and work history [48]. Raters used this assessment to gather information on poverty levels as well as presence of caregivers in the participant’s life.

### Data Analyses

Unless otherwise specified, SPSS (version 20) software was used for analyses. Cronbach’s alpha was used to assess the internal consistency of the TCL 2.0. In addition, intraclass correlation coefficients (ICCs) were used to examine inter-rater reliability. ICC estimates were calculated using a two-way random effects model on average measures with absolute agreement. In line with our previous trauma report [39], principal component analysis (PCA) with Varimax rotation was used to examine the dimensionality of the TCL 2.0. Trauma categories that had loadings of ≥ 0.5 were assigned to each principal component (PC; [58]). In addition, this PCA was followed by exploratory structural equation modeling (ESEM) to rigorously test how well a two-component solution accounted for the TCL 2.0 data. This was done for a few reasons. First, the PCA approach, one of the most frequently employed statistical methods, is often used to summarize a main component in a dataset, but it assumes perfect measurement (i.e., does not model residual error/unique variance). ESEM, like confirmatory factor analysis, estimates common factor variance separately from error/unique variance, and therefore it provides a riskier test of model fit. In other words, PCA is simply used to reduce or summarize the data, whereas ESEM is designed to account *for* the data [59]. Also, PCA can produce problematic solutions when the number of variables per factor are low [60]. Therefore, subsequent ESEM after PCA provides a robust test of the theoretical model (i.e., two different latent variables reflecting trauma experiences). Mplus software was used for ESEM analyses [61], and standard fit criteria (i.e., CFI > 0.90; RMSEA < 0.08) were used to assess ESEM fit (see [62] for a discussion on ESEM approach). Given the ordinal nature of the TCL domains, the robust weighted least squares estimator was used for ESEM and efficient rotation was done via GEOMIN (oblique rotation; Mplus default). Using standardized factor loadings and residual error/unique variance, Omega reliability estimates were calculated in terms of common factor variance over total variance [63, 64]. More specifically, Omega was calculated from Mplus standardized model parameters in terms of the common sources of unit-weighted total score variance (i.e., sum of the squared loadings), divided by the unit-weighted total score variance (common sources of total score variance plus error/unique variance). Additionally, we used Pearson correlations to examine the relationships between the TCL 2.0 total and component scores with a self-reported trauma measure (i.e., the UES), measures of psychopathology obtained via the K-SADS (i.e., anxiety, mood, and PTSD), and psychopathic traits assessed via the PCL:YV.

Finally, multivariate Cox proportional-hazard regressions were run using the survival package [65] in R [66] to test whether TCL 2.0 scores predicted time (in months) to first felony-related re-offense. New Mexico recidivism data were obtained from the Center for Science and Law’s Criminal Record Database (CRD; [67]). More details are provided in [68]; briefly, re-offense data was extracted from the CRD of criminal court records for offenders in New Mexico. Data in the CRD were matched to current participants via four separate identifiers (i.e., first and last name, date of birth, and social security number). Extensive online searches including social media, White Pages, Been Verified, county records, New Mexico Corrections Department offender search, and out–of–state inmate databases were conducted for the entire sample. This enabled us to compile recidivism data for subjects who were not found in the CRD. Felony recidivism was operationally defined as any felony re-offense following the participant’s release from the juvenile correctional facility. This recidivism data was examined continuously (i.e., time [in months] to first felony re-offense following release outcomes) via multivariate Cox proportional-hazard regressions. Consistent with other studies from our research group [68, 69], time at risk was defined as the time period between an individual’s release date and their felony re-offense date or the end of the follow up window (August 31, 2019) for those who did not commit a felony offense. In participants with all available data (i.e., *n* = 221 offenders), ~ 74% of our participants (i.e., *n* = 164) committed a felony offense following their release from the juvenile correctional facility. One-tailed statistical tests were performed, given that trauma has been previously associated with recidivism in existing studies [20, 21]. In each model, TCL 2.0 scores (either the total trauma score or the two PC scores) were entered along with IQ scores and PCL:YV factor scores, to see if TCL 2.0 scores remained associated with time to felony re-offense. Because IQ and PCL:YV scores have been previously linked to criminal behavior [49, 50], we accounted for shared variance with these relevant covariates in the Cox proportional-hazard regression analyses.

## Results

### Descriptive Statistics

**Fig. 1:**
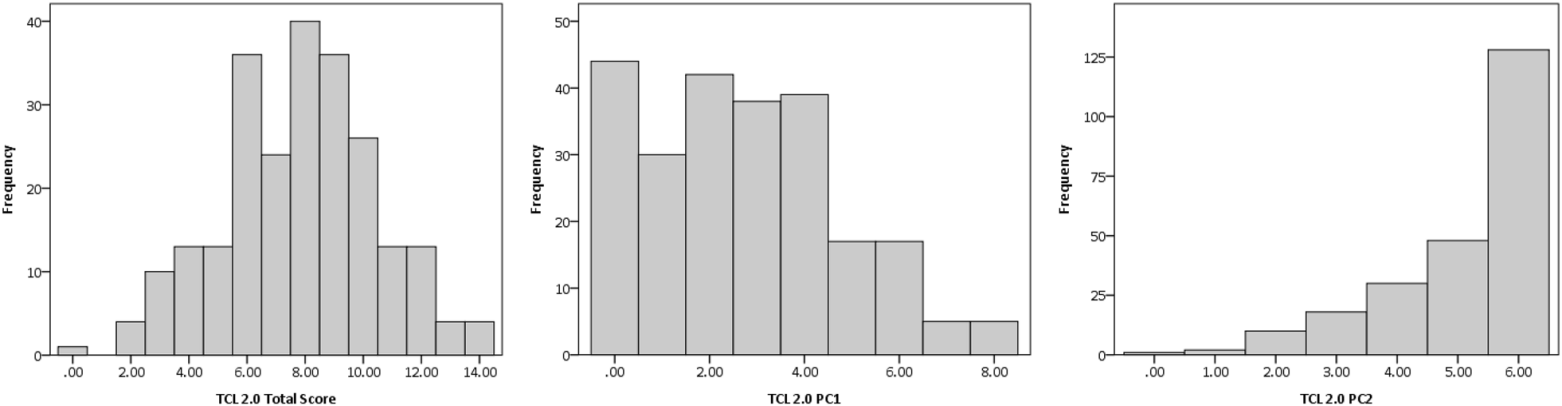
TCL 2.0 Histogram Plots. Note. TCL 2.0 PC1 refers to TCL 2.0 Experienced Trauma scores; TCL 2.0 PC2 refers to TCL 2.0 Community Trauma scores

A visual representation of TCL 2.0 scores is provided in Fig. 1. Overall, participants included in the current study were characterized by high rates of trauma. The highest possible TCL 2.0 total score is 14, and the total possible score for each trauma category is two. In this sample, participants largely reported experiencing high rates of Community Violence, Observed Trauma, Traumatic Loss, and Neglect/Poverty, and lower rates of Emotional Abuse, Physical Abuse, and Sexual Abuse. High scores were also found when incorporating chronicity scores. The total possible score for the TCL 2.0 Chronicity score is 42, and the total possible chronicity score for each trauma category is six (see Table 1 for descriptive statistics for all TCL 2.0 scores in the sample).

**Table 1:** Descriptive Statistics for TCL 2.0, PCL: YV, CPSS, and UES

Variable	M	SD	Range	Skewness
TCL 2.0 Total	7.81	2.65	0–14	−0.09
TCL 2.0 PC1	2.73	2.06	0–8	0.46
TCL 2.0 PC2	5.08	1.26	0–6	−1.40
Emotional Abuse	0.45	0.73	0–2	1.29
Physical Abuse	0.95	0.90	0–2	0.11
Sexual Abuse	0.29	0.67	0–2	2.01
Neglect/Poverty	1.04	0.83	0–2	−0.08
Community Violence	1.75	0.56	0–2	−2.14
Observed Trauma	1.69	0.62	0–2	−1.82
Traumatic Loss	1.65	0.65	0–2	−1.61
TCL 2.0 Chronicity	14.46	6.53	0–35	0.33
Chronicity PC1	5.37	4.47	0–19	0.64
Chronicity PC2	9.09	3.31	0–17	−0.25
Emotional Abuse Chronicity	0.89	1.63	0–6	1.90
Physical Abuse Chronicity	1.94	2.11	0–6	0.70
Sexual Abuse Chronicity	0.35	0.87	0–6	3.13
Neglect/Poverty Chronicity	2.19	2.02	0–6	0.45
Community Violence Chronicity	2.94	1.40	0–6	−0.18
Observed Trauma Chronicity	3.47	1.91	0–6	−0.17
Traumatic Loss Chronicity	2.68	1.51	0–6	0.12
PCL:YV Total	23.55	6.11	2–35	−0.35
PCL:YV Factor 1	6.67	3.11	0–15	0.34
PCL:YV Factor 2	14.63	3.24	1–20	−1.12
CPSS (*n* = 76)	11.11	9.85	0–43	0.79
UES (*n* = 86)	7.36	5.23	0–25	1.18

TCL 2.0 total refers to the total trauma score assessed via the TCL 2.0 (α = 0.57); TCL 2.0 PC1 refers to Experienced Trauma scores; TCL 2.0 PC2 refers to Community Trauma scores; TCL 2.0 Chronicity refers to the TCL 2.0 Chronicity score; Chronicity PC1 refers to Experienced Trauma Chronicity scores; Chronicity PC2 refers to Community Trauma Chronicity scores; PCL:YV Total, Factor 1, and Factor 2 scores refer to total, Factor 1 (interpersonal and affective psychopathic traits), and Factor 2 (lifestyle/behavioral and antisocial/developmental psychopathic traits); CPSS refers to the Childhood PTSD Symptoms total score; UES refers to Upsetting Events Survey total score

### Inter-Rater Reliability

Inter-rater reliability for the seven different trauma categories was assessed using ICCs. Similar to our previous report [39], moderate to high ICCs were observed between raters for individual trauma categories: Emotional Abuse (ICC = 0.72), Physical Abuse (ICC = 0.85), Sexual Abuse (ICC = 0.83), Neglect/Poverty (ICC = 0.68), Community Violence (ICC = 0.75), Observed Trauma (ICC = 0.70), and Traumatic Loss (ICC = 0.72). Additionally, high ICCs were observed for the TCL 2.0 Total Trauma Score (ICC = 0.83).

### Dimensionality Reduction

PCA of the TCL 2.0, with Varimax rotation, yielded a two-component solution, accounting for 47% of the variance in the total score. Based on our previous method [39], this two-component solution was determined by setting the Eigenvalue threshold to greater than one. As reported in Table 2, Emotional Abuse, Physical Abuse, Sexual Abuse, and Neglect/Poverty loaded onto PC1 (i.e., Experienced Trauma), whereas Community Violence, Observed Trauma, and Traumatic Loss loaded onto PC2 (i.e., Community Trauma). Observed Trauma loaded onto both PC1 and PC2 (i.e., component loadings > 0.4 for both components); however, since this trauma category loaded more strongly onto PC2 (0.581) compared to PC1 (0.424), we considered Observed Trauma to be an item included within PC2 rather than PC1. The ESEM results using GEOMIN (oblique) rotation provided strong support for a two-component TCL 2.0 model (CFI = 1.0; RMSEA = 0.00; 90%CI = 0.00 – 0.03; for factor loadings and standard errors, please see Table 3). Also, standardized loadings from the ESEM can be used to generate Omega reliability estimates [70], which were acceptable for both TCL PC1 (0.70) and PC2 (0.65), given the small number of items per component. The average TCL 2.0 PC1 score was 2.73 (*SD* = 2.06, range: 0 – 8, α = 0.56) and the average TCL 2.0 PC2 score was 5.08 (*SD* = 1.26, range: 0 – 6, α = 0.45). Finally, TCL 2.0 component scores were characterized by high inter-rater reliability (i.e., PC1 ICC = 0.86, PC2 ICC = 0.77).

**Table 2:** Principal Component Loadings

Type of Trauma	PC1 (Experienced)	PC2 (Community)
Emotional Abuse	**0.643**	0.025
Physical Abuse	**0.761**	0.097
Sexual Abuse	**0.570**	−0.212
Neglect/Poverty	**0.580**	0.211
Community Violence	0.041	**0.766**
Observed Trauma	0.424	**0.581**
Traumatic Loss	−0.093	**0.647**

Principal component analysis (PCA) with Varimax rotation was used to examine the component solution of the TCL 2.0. Individual trauma categories that had loadings of ≥ 0.5 were assigned to a principal component (PC), with emotional abuse, physical abuse, sexual abuse, and neglect/poverty loading onto PC1 (Experienced Trauma) and community violence, observed trauma, and traumatic loss loading onto PC2 (Community Trauma)

Individual trauma categories that had loadings of ≥ 0.5 were assigned to a PC and appear here in bold

**Table 3:** ESEM Loadings and Standard Errors

TCL 2.0 Domain	TCL 2.0 PC1		TCL 2.0 PC2	
	Loading	SE	Loading	SE
Emotional Abuse	0.61	0.09	0.02^*ns*^	0.12
Physical Abuse	0.82	0.09	−0.01^*ns*^	0.02
Sexual Abuse	0.50	0.11	−0.16^*ns*^	0.14
Neglect/Poverty	0.48	0.09	0.13^*ns*^	0.11
Community Violence	0.00^*ns*^	0.01	0.78	0.19
Observed Trauma	0.45	0.13	0.56	0.17
Traumatic Loss	−0.02^*ns*^	0.12	0.39	0.13

All factor loadings are significant (p’s < 0 .001), unless designated *ns*

### Correlation Analyses

TCL 2.0 total trauma scores were significantly positively correlated with TCL 2.0 PC1 scores (*r* = 0.89, *p* < 0.001) and PC2 scores (*r* = 0.65, *p* < 0.001). TCL 2.0 PC1 and PC2 scores were significantly positively correlated with each other (*r* = 0.22, *p* = 0.001), as expected using GEOMIN (oblique) rotation during ESEM. Additionally, TCL 2.0 total trauma scores were significantly positively correlated with total scores derived from a self-report measure of childhood trauma, assessed via the UES total score (*r* = 0.34, *p* = 0.002).

Regarding external measures for anxiety disorders, mood disorders, and PTSD, findings were similar to our prior study [39]. For example, TCL 2.0 total and PC2 scores were not significantly correlated with diagnoses for anxiety disorders (TCL 2.0 total *r* = 0.00, *p* = 0.972; PC2 *r* = -0.10, *p* = 0.145) or mood disorders (TCL 2.0 total *r* = 0.09, *p* = 0.165; PC2 *r* = -0.08, *p* = 0.243) in the current study. Additionally, while TCL 2.0 PC1 scores were not significantly correlated with diagnoses for anxiety disorders (*r* = 0.06, *p* = 0.350), these scores were significantly correlated with diagnoses for mood disorders (*r* = 0.17, *p* = 0.012). In contrast, and inconsistent with our previous report, TCL 2.0 total trauma and component scores were not significantly correlated with PTSD diagnoses (TCL 2.0 total *r* = 0.06, *p* = 0.385; PC1 *r* = 0.05, *p* = 0.482; PC2 *r* = 0.05; *p* = 0.500). This may relate to the low base rate of PTSD observed in the current sample (*n* = 16, 6.8% of the sample) compared to our previous study (*n* = 26, 25%). Supporting this notion, TCL 2.0 total trauma scores were significantly correlated with CPSS self-reported PTSD symptomatology in a subsample of participants with available scores (*n* = 76; *r* = 0.24, *p* = 0.040).

Furthermore, TCL 2.0 scores were significantly positively correlated with PCL: YV total and factor scores. For example, TCL 2.0 total trauma scores were significantly positively correlated with PCL: YV total (*r* = 0.29, *p* < 0.001), Factor 1 (*r* = 0.18, *p* = 0.004), and Factor 2 (*r* = 0.31, *p* < 0.001) scores. Additionally, TCL 2.0 PC1 scores were significantly positively correlated with PCL: YV Factor 1 (*r* = 0.18, *p* = 0.005) and Factor 2 (*r* = 0.21, *p* = 0.001) scores, whereas TCL 2.0 PC2 scores were significantly positively correlated with PCL: YV Factor 2 scores (*r* = 0.30, *p* < 0.001), but not PCL: YV Factor 1 scores (*r* = 0.09, *p* = 0.175) (see Table 4).

**Table 4:** Correlations between TCL 2.0 Scores, Measures of Psychopathology, Psychopathic Traits, and Additional Trauma Measures

	1	2	3	4	5	6	7	8	9	10	11	12	13	14	15	16	17	18
1. TCL 2.0 Total	–	**0.56**	**0.69**	**0.44**	**0.61**	**0.41**	**0.58**	**0.35**	**0.89**	**0.65**	0.06	0.00	0.09	**0.29**	**0.18**	**0.31**	**0.34**	**0.24**
2. Emotional Abuse			**0.34**	**0.18**	**0.19**	0.02	**0.21**	0.06	**0.63**	**0.14**	0.05	0.07	**0.23**	0.05	0.02	0.07	0.12	**0.31**
3. Physical Abuse				**0.24**	**0.34**	0.10	**0.29**	0.01	**0.77**	**0.19**	0.02	0.12	0.09	**0.27**	**0.23**	**0.25**	0.18	0.13
4. Sexual Abuse					**0.14**	0.01	0.09	−0.05	**0.55**	0.02	0.09	0.08	0.11	0.02	0.05	−0.01	0.20	**0.31**
5. Neglect/Poverty						0.11	**0.23**	0.07	**0.66**	**0.20**	−0.01	−0.10	0.02	**0.21**	**0.14**	**0.21**	0.09	−0.03
6. Community Violence							**0.32**	**0.21**	0.10	**0.71**	−0.00	−0.11	**−0.13**	**0.23**	0.11	**0.29**	0.19	0.11
7. Observed Trauma								0.13	**0.32**	**0.70**	0.05	−0.08	0.02	0.09	0.06	0.11	**0.22**	0.05
8. Traumatic Loss									0.04	**0.67**	0.04	−0.02	−0.05	**0.17**	0.02	**0.23**	**0.27**	0.02
9. TCL 2.0 PC1										**0.22**	0.05	0.06	**0.17**	**0.22**	**0.18**	**0.21**	**0.22**	**0.25**
10. TCL 2.0 PC2											0.05	−0.10	−0.08	**0.23**	0.09	**0.30**	**0.33**	0.07
11. PTSD												0.05	**0.18**	0.03	−0.00	0.05	0.10	**0.31**
12. Anxiety													**0.18**	−0.00	−0.04	0.03	−0.03	0.19
13. Mood Disorder														−0.07	−0.05	−0.06	−0.10	0.11
14. PCL:YV Total															**0.85**	**0.87**	**0.34**	0.08
15. PCL:YV Factor 1																**0.51**	**0.26**	0.02
16. PCL:YV Factor 2																	**0.30**	0.16
17. UES Total																	–	**0.30**
18. CPSS Total																		–

TCL 2.0 total refers to the total trauma score assessed via the TCL 2.0; TCL 2.0 PC1 refers to TCL 2.0 Experienced Trauma scores; TCL 2.0 PC2 refers to TCL 2.0 Community Trauma scores; PTSD, Anxiety, and Mood Disorder refer to diagnoses for PTSD, anxiety, and mood disorders obtained from the K-SADS; PCL:YV total, Factor 1, and Factor 2 scores refer to total, Factor 1 (interpersonal and affective psychopathic traits), and Factor 2 (lifestyle/behavioral and antisocial/developmental psychopathic traits); UES total refers to the total score derived from the Upsetting Events Survey; CPSS total refers to the total score derived from the Childhood PTSD Symptoms Scale. Significant correlations (*p* < .05) are highlighted in bold

### Multivariate Cox Proportional-Hazard Regression Analyses

As reported in Table 5, when including IQ and PCL:YV factor scores in the multivariate model, TCL 2.0 total trauma scores were not significantly associated with time to felony re-offense (Model 1). However, TCL 2.0 PC2 scores (Community Trauma) were significantly associated with time to felony re-offense (β = 0.16, *p* = 0.018), even when including TCL 2.0 PC1 scores (Experienced Trauma), IQ, and PCL:YV factor scores in the model (Model 2).

**Table 5:** Multivariate Cox Proportional-Hazard Regression Analyses

Variable	β	exp (β)	SE (β)	p-value
**Model 1**				
IQ	−0.02	0.98	0.01	0.003**
PCL: YV Factor 1	0.02	1.02	0.03	0.301
PCL: YV Factor 2	0.12	1.13	0.03	< 0.001**
TCL 2.0 Total	0.03	1.03	0.03	0.164
**Model 2**				
IQ	−0.02	0.98	0.01	0.006**
PCL:YV Factor 1	0.03	1.03	0.03	0.198
PCL:YV Factor 2	0.11	1.11	0.03	0.001**
TCL 2.0 PC1	−0.01	0.99	0.04	0.382
TCL 2.0 PC2	0.16	1.18	0.08	0.018**
**Model 3**				
TCL 2.0 Total	0.04	1.04	0.05	0.211
UES total	0.03	1.03	0.02	0.086
**Model 4**				
TCL 2.0 PC1	−0.08	0.92	0.07	0.112
TCL 2.0 PC2	0.43	1.54	0.14	0.001**
UES Total	0.02	1.02	0.02	0.165

IQ refers to full-scale IQ scores obtained from the WAIS-III or WISC-IV; PCL:YV Factor 1 and 2 scores refer to factor scores (i.e., Factor 1: interpersonal and affective psychopathic traits; Factor 2: lifestyle/behavioral and antisocial/developmental psychopathic traits) derived from the PCL:YV; TCL 2.0 total refers to the total trauma score obtained from the TCL 2.0; TCL PC1 and PC2 scores refer to component scores obtained from the TCL 2.0, with PC1 representing Experienced Trauma scores and PC2 representing Community Trauma scores; UES total refers to the total score derived from the Upsetting Events Survey. Significant effects (*p* < .05) based on a one-tailed statistical test are highlighted using asterisks (**)

Additional multivariate Cox proportional-hazard regression analyses were performed including TCL 2.0 total and component scores along with total scores from a self-report measure of childhood trauma (i.e., the UES). Here, we investigated whether the TCL 2.0, the UES, or both measures, were associated with time to felony re-offense. As shown in Table 5, when including TCL 2.0 total trauma scores along with UES total scores, neither trauma measure was significantly associated with felony re-offense outcomes (Model 3). However, when including TCL 2.0 component scores along with UES total scores, TCL 2.0 PC2 scores (Community Trauma), but not UES scores, were associated with time to felony re-offense (β = 0.43, *p* = 0.001; Model 4).

## Discussion

The goal of this study was to expand upon our previously developed trauma assessment, with the development of the TCL 2.0, and to examine its psychometric properties. Compared to our earlier version of the TCL [39], the revised TCL 2.0 provides raters with additional resources and more detailed instructions for scoring, and it includes age bins to gather more detail on when traumatic events occurred across childhood. The age bin data allow raters to distinguish between individuals who have experienced a few isolated traumatic events versus those who have experienced chronic, continuous trauma across their lifespan. Here, we determined the component solution of the TCL 2.0 and examined correlations between TCL 2.0 components and total trauma scores and external variables, including psychopathology (i.e., rates of anxiety, depression, PTSD), psychopathic traits, and self-report measures of PTSD and trauma. Finally, due to the established link between early childhood trauma and antisocial outcomes [22, 71, 72], we examined whether TCL 2.0 scores were associated with time to felony re-offending via multivariate Cox proportional-hazard regression analyses.

This sample was characterized by elevated scores on the TCL 2.0, indicating that these high-risk youth had experienced significant trauma during their childhood. Amongst the various trauma categories, participants scored highest in community violence, observed trauma, and traumatic loss, whereas participants scored lowest in sexual abuse. Agreement between raters was “substantial” for most trauma categories, with the raters being in “almost perfect agreement” for physical abuse, sexual abuse, and the TCL 2.0 total trauma score [73]. Via PCA and ESEM, the TCL 2.0 was found to have a two-component solution, whereby emotional abuse, physical abuse, sexual abuse, and neglect/poverty loaded onto one component (PC1: Experienced Trauma), and community violence, observed trauma, and traumatic loss loaded onto another component (PC2: Community Trauma). In addition, the TCL 2.0 total trauma scores and component scores were positively correlated with a self-report measure of trauma (i.e., the UES), whereas TCL 2.0 total trauma scores and PC1 scores were positively correlated with self-reported PTSD symptomatology via the CPSS. PC1 (Experienced Trauma) was positively correlated with mood disorder diagnoses. Finally, TCL 2.0 total trauma scores and PC1 scores were positively correlated with PCL: YV total and factor scores. PC2 (Community Trauma) was positively correlated with PCL: YV total and Factor 2 scores. Because the correlation between the TCL 2.0 and other measures (i.e., PCL: YV and K-SADS) was not so high as to suggest redundancy, extracting trauma-related information from these assessments separately from their usual use is supported.

Several similarities emerged between our current findings and those included in our previous report. In both studies, the TCL total score and component scores are positively correlated with other self-report measures of trauma, suggesting the concurrent validity of the TCL (i.e., both the original TCL and the TCL 2.0). In addition, raters achieved a similarly high degree of inter-rater reliability in both studies. Finally, we examined prevalence rates of trauma. Out of our current sample of *n* = 237 incarcerated male youth, we found that 99.58% of individuals scored at least a two or higher on the TCL 2.0 total trauma score, which is consistent with our previous report. Similar rates of trauma in comparable New Mexico samples were reported in other studies, where more than 99% of the sample reported at least one ACE [51]. TCL 2.0 rates of physical abuse (37.55%) and neglect/poverty (36.71%) were also consistent with our prior study. While there were multiple similarities between our current and prior report, some discrepancies also emerged. For example, rates of emotional abuse and sexual abuse were lower in the current sample, while rates of community violence, observed trauma, and traumatic loss were higher. Though assessing incarcerated youth in both reports, differences relating to sample composition may explain some of the differences observed in the current results. For example, perhaps youth from Wisconsin undergo more Experienced Trauma (PC1) compared to youth from New Mexico, who exhibit higher rates of Community Trauma (PC2). Additionally, differences relating to race and ethnicity composition of the samples may have contributed to disparate results obtained between reports.

In addition, there are some differences to note between the two studies with regard to TCL correlations to PTSD diagnoses, mood disorder diagnoses, and psychopathic traits. First, based on the relationship between childhood trauma and the development of PTSD [74], we expected to find that the TCL 2.0 would be positively correlated with PTSD diagnoses. However, this was not observed, which is likely due to the low base rate of participants who met criteria for PTSD in this sample (i.e., 6.8% of the sample). This finding is similar to other studies, where samples with high traumatic event exposure have low PTSD prevalence [75], and therefore the low base rate of PTSD in our current sample is not indicative of low trauma exposure. That said, the TCL 2.0 was significantly positively correlated with the CPSS, a self-report measure of PTSD. Second, while no relationship between the TCL and mood disorder diagnoses was found in our original report, here we report that PC1 (Experienced Trauma) was positively correlated with mood disorder diagnoses. This difference is likely due to the smaller sample size in our initial report. However, the relationship between trauma and mood disorders we find here is consistent with other reports, indicating that childhood trauma is linked to the development (and persistence) of mood disorders in both adolescence and adulthood [76–78]. Third, in our initial report, we observed negative correlations between the TCL and psychopathic traits, whereas in our current sample we report positive correlations between the TCL 2.0 and psychopathic traits. The difference in the relationship between trauma and psychopathy in our two reports could stem from the differences in trauma exposure between our two samples. However, it should be noted that the results of our current report are similar to other studies that show a positive relationship between trauma and psychopathic traits [55, 57, 79].

The TCL component structure also differed slightly between our current and previous report. While PC1 (Experienced Trauma) was the same across reports, our previous report found a three-component solution, where community violence and observed trauma loaded onto the second component, and traumatic loss loaded onto a third component. However, for the TCL 2.0, we observed a two-component solution, with community violence, observed trauma, and traumatic loss all loading onto PC2 (Community Trauma), and these results were robustly supported by the ESEM results. This difference is likely due to the smaller sample size included in our original report, as component structure replicability can depend on the sample size of the initial study [80], though it may also be due in part to the modifications made to the TCL assessment (specifically with regard to traumatic loss scoring and instructions).

In addition to noting similarities and differences between the current report and our previous study, here, we expanded upon our previous report by investigating whether TCL 2.0 scores were associated with future antisocial outcomes, including felony re-offending. When performing multivariate Cox proportional-hazard regression analyses, higher TCL 2.0 PC2 scores (Community Trauma) were associated with a faster time to felony re-offense. These scores were associated with felony re-offense outcomes, even when including TCL 2.0 PC1 scores (Experienced Trauma), IQ, and PCL: YV factor scores in the model. Additionally, we investigated whether two different measures of childhood trauma (i.e., the TCL 2.0 and the UES) were each, or uniquely, associated with re-offense outcomes. While the UES was not significantly associated with time to felony re-offense, TCL 2.0 PC2 scores (Community Trauma) did emerge as a significant predictor of such outcomes, with higher scores being associated with a faster time to felony re-offense. Such results are consistent with previously published studies suggesting that a prior history of childhood trauma is associated with recidivism [22, 71, 72]. These results also suggest that trained-rater trauma assessments (i.e., the TCL 2.0) may be uniquely associated with re-offense outcomes among incarcerated individuals compared to self-report trauma measures (i.e., the UES). Additionally, our results suggest that a specific *type* of trauma (i.e., Community Trauma versus Experienced Trauma) may be uniquely associated with felony re-offense outcomes. Because Community Trauma often involves exposure to violence in one’s family or community (e.g., getting jumped or witnessing abuse or death of a friend or family member), higher scores may suggest that a juvenile was more immersed in a high-risk and violent environment prior to incarceration, which may increase their risk for serious re-offending, including committing felony offenses. Furthermore, returning to high-risk and violent environments post-incarceration could lead to continued trauma exposure and thus increase the risk of felony recidivism.

Due to the many limitations of self-report trauma assessments, the trained-rater TCL 2.0 is a valuable tool for measuring childhood trauma. Relying fully on self-report instruments to gather data about childhood trauma history can be potentially problematic and lead to inaccurate reports. As the TCL 2.0 is scored by a trained rater, the data gathered in this assessment is less likely to be characterized by these inaccuracies. Supporting this notion, we observed low classification agreement [54] between the TCL 2.0 and UES (Cohen’s kappa = 0.30, *p* = 0.005), suggesting that with the UES, individuals may be normalizing their experience or underreporting trauma history. Additional work will be needed to test whether our results in this report extend to female and adult samples. Furthermore, while an institutional file is not strictly necessary to score the TCL 2.0, it does provide additional information about traumatic experiences. Thus, the TCL 2.0 may be best utilized to score trauma histories in forensic or institutionalized populations, where records are readily available for reference when scoring.

## Conclusions

In summary, the TCL 2.0 is uniquely situated to comprehensively assess difficult-to-measure and highly relevant childhood trauma categories in forensic populations. Due to the established relationship between trauma history and antisocial behavior, it is of utmost importance to provide individuals with appropriate trauma-informed interventions, ideally before they start committing crimes. Because the trained-rater TCL 2.0 assessment has predictive utility with regard to antisocial outcomes (i.e., time to felony re-offense), this assessment could be beneficial as a screening tool in at-risk youth to help provide them with the trauma intervention they need. Furthermore, given that the TCL 2.0 has predictive utility for felony re-offense, this assessment can also be useful in incarcerated populations to provide individuals with appropriate trauma-informed treatment prior to their release to reduce the risk of re-offense.

## Data Availability

Due to the potential for re-identification of study participants in this sensitive population (i.e., incarcerated juveniles), data presented in this manuscript are not readily available. To obtain data used in this manuscript, which may be shared under a data use agreement, contact Dr. Kent Kiehl (kkiehl@mrn.org).

## Abbreviations

ACE: Adverse childhood experience
CPSS: Childhood PTSD symptoms scale
CRD: Criminal record database
CTQ: Childhood Trauma Questionnaire
ESEM: Exploratory structural equation modeling
ICC: Intraclass correlation coefficient
IQ: Intelligence quotient
JVQ: Juvenile Victimization Questionnaire
K-SADS: Kiddie-schedule for affective disorders and schizophrenia
MAYSI-2: Massachusetts Youth Screening Instrument-Version 2
NICHD: National Institute of Child Health and Human Department
NIMH: National Institute of Mental Health
OHRP: Office for Human Research Protections
PC: Principal component
PCA: Principal component analysis
PCL: YV: Psychopathy Checklist: Youth Version
PHQ: Post-head Injury Symptoms Questionnaire
PTSD: Post-traumatic stress disorder
SES Questionnaire: Socioeconomic Status Questionnaire
TCL: Trauma Checklist
TCL 2.0: Trauma Checklist 2.0
UES: Upsetting Events Survey
US: United States
WAIS-III: Wechsler Adult Intelligence Scale 3rd edition
WISC-IV: Wechsler Intelligence Scale for Children 4th edition
